# Irreversible Electroporation in Patients with Pancreatic Cancer: How Important Is the New Weapon?

**DOI:** 10.1155/2018/5193067

**Published:** 2018-04-26

**Authors:** Guo Tian, Xueping Liu, Qiyu Zhao, Danxia Xu, Tian'an Jiang

**Affiliations:** ^1^Department of Ultrasound Medicine, the First Affiliated Hospital, Zhejiang University School of Medicine, Hangzhou, China; ^2^Key Laboratory of Precision Diagnosis and Treatment for Hepatobiliary and Pancreatic Tumor of Zhejiang Province, Hangzhou, China; ^3^Department of Ultrasound, People's Hospital, Quzhou, China

## Abstract

**Background:**

Pancreatic cancer (PC) is a deadly disease with poor prognosis in the general population. We aimed to quantitate overall survival of patients with PC after irreversible electroporation (IRE) and the incidence of relevant complications.

**Methods:**

We performed a literature search via five electronic databases (PubMed, Embase, Web of Science, Scopus, and Cochrane Library databases) up to August 2017. The primary outcomes were overall survival and prognosis. Secondary outcomes included the response of post-IRE complications. Fixed-effects or random-effects meta-analysis was conducted to pool these data.

**Results:**

A total of 15 eligible articles involving 535 patients were included. The primary outcomes showed that the pooled prevalence estimates of overall survival were 94.1% (95% CI: 90.7–97.5), 80.9% (95% CI: 72.5–89.4), 54.5% (95% CI: 38.3–70.6), and 33.8% (95% CI: 14.2–53.5) at 3, 6, 12, and 24 months, and the pooled prevalence data of complete response (CR) at 2 months, partial response (PR) at 3 months, and progression at 3 months were 12.5% (95% CI: 2.9–22.2), 48.5% (95% CI: 39.4–57.6), and 19.7% (95% CI: 7.3–32.2), respectively. The secondary outcomes showed that the pooled prevalence values of post-IRE complications were abscess 6.6% (95% CI: 0.2–13), fistula 10.6% (95% CI: 2.5–18.7), pain 33.5% (95% CI: 14.5–52.5), infection 16.1% (95% CI: 3.9–28.4), thrombosis 4.9% (95% CI: 1.2–8.5), pancreatitis 7.2% (95% CI: 3.1–11.2), bleeding 4.2% (95% CI: −0.5–8.9), cholangitis 4.2% (95% CI: −0.5–8.9), nausea 9.6% (95% CI: 4.4–14.8), biliary obstruction 13.8% (95% CI: 4.2–23.3), chest tightness 7.6% (95% CI: 0.5–14.6), and hypoglycemia 5.9% (95% CI: −0.4–12.2).

**Conclusions:**

This meta-analysis indicated a clear survival benefit for PC patients who received irreversible electroporation therapy, although future safety and effectivity monitoring from more large-scale studies will be needed.

## 1. Introduction

Pancreatic cancer is a highly malignant disease with poor prognosis, which accounts for about 4% of cancer-related deaths in general population [1, 2]. Although surgical resection provides chance for curable treatment, about only twenty percent of patients were resectable [3]. In recent decades, interventional therapies such as high-intensity focused ultrasound (HIFU) [4, 5], radiofrequency ablation (RFA) [6, 7], and microwave ablation (MWA) [8] have become fine ways in the treatment of pancreatic tumors.

Unlike those methods, the emerging family member in ablative techniques is irreversible electroporation (IRE), which is an invasive nonthermal ablation method by utilizing short direct current pulses to increase cell membrane permeability and result in cellular death [9]. Animal model showed that IRE produced the clear borders between treated and untreated area [10]. It would not be affected by the heat sink and suitable for lesions in the intricate regions. In recent decades, this technique has been generally reported in substantial number of tumor tissues located in liver [11, 12], pancreas [13, 14], lung [15], and kidney [16, 17], where IRE seemed to be effective. However, existing clinical experiences and sample size were still insufficient. In this regard, thus we performed the systematic review to estimate the efficacy and safety of IRE treatment for patients with PC in terms of overall survival and the complications.

## 2. Methods

### 2.1. Search Strategy and Study Eligibility

The systematic review was conducted based on the Preferred Reporting Items for Systematic Reviews and Meta-Analyses (PRISMA) guidelines [18]. We cautiously did a literature search through five electronic databases (PubMed, Embase, Web of science, Scopus, and Cochrane Library databases) from inception to August 2017. Subject headings and keywords of irreversible electroporation and pancreatic cancer were retrieved (Supplementary Text). The retrieved references were managed using Endnote X7, and duplicates were filtered through the software.

### 2.2. Inclusion Criteria

Articles would be included when they met the following criteria: (1) they were published original reports; (2) they focused on IRE treatments of patients with PC; (3) they reported the overall survival, prognosis, or the IRE-related complications; and (4) follow-up interval was at least 1 month after IRE therapies. Literatures searched had no language restrictions. Systematic reviews, meta-analyses, conference presentations, and letters were excluded. All disagreements of opinion in this study were discussed and resolved by consensus with the third arbiter.

### 2.3. Data Extraction and Quality Assessment

At first, two individuals independently classified all literatures using the established protocols. The initial classification was evaluated by the third reviewer to maximize its accuracy. Then full-text papers were cross-checked by two investigators for further analysis according to inclusion criteria. Finally, two authors independently extracted the data from included studies using predefined protocols, such as author, study period, design style, country, population characteristics, tumor size, treatment methods, patients, gender, age, follow-up interval, complication, imaging methods, and prognosis. The primary outcomes were overall survival at 3-, 6-, 12- and 24-month time points and complete response at 2 months, partial response at 3 months, and progression at 3 months, and secondary outcomes included the incidence of post-IRE complications (abscess, fistula, pain, infection, thrombosis, pancreatitis, bleeding, cholangitis, nausea, biliary obstruction, chest tightness, and hypoglycemia).

We rated the quality of each subgroup as high, moderate, low, or very low based on the Grading of Recommendations Assessment, Development, and Evaluation (GRADE) guideline. Randomized controlled trials had an initial high evidence quality and were downgraded if present in the risk of bias, inconsistency, indirectness, imprecision, or publication bias, whereas observational studies began with a low evidence quality and may be upgraded in terms of large effect, plausible confounding, or dose-response gradient [19].

The pooled incidence rates of overall survival and complications were calculated to combine the summary result from each subgroup. *I*^2^ statistics, chi-square test (*χ*^2^), and *τ*^2^ were used for the assessment of statistical between-study heterogeneity. *p* value of 0.05 or less indicated statistical significance. If *I*^2^ values less than 50% were considered little heterogeneity, then a fixed-effect model was used. Otherwise, a random-effects model was performed for substantial heterogeneity [20]. In this study, sensitivity analysis was to check the impact of each study on the whole incidence assessment by consecutively deleting each study. Bias to small study effects was quantitatively estimated by the Egger test [21]. All analysis in this study was performed using Stata 12.0 software.

## 3. Results

### 3.1. Study Characteristics

**Figure 1:**
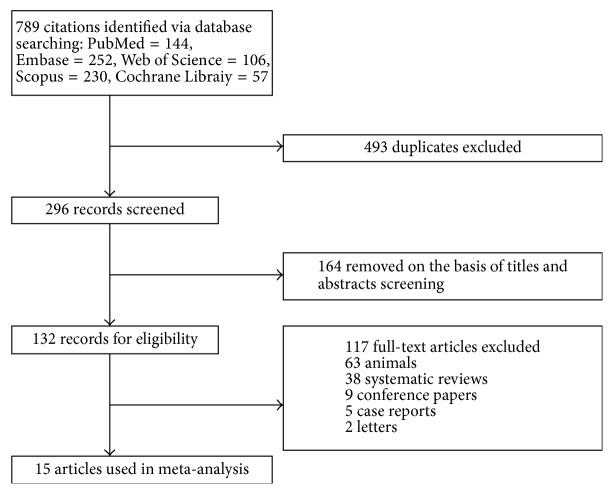
Study selection process.

A total of 789 articles were identified through those five databases, of which 774 records were duplicates and removed based on predefined inclusion criteria. Finally, a total of 15 studies related to IRE and PC were included (Figure 1) [9, 13, 22–34]. These studies involved 535 cases (female 46.5%), in which the mean age of participants was ranging from 53 to 69 years, and there were 8 studies conducted in Europe, 4 in Asia, and 3 from North America. These were all described in Table 1. The risk of methodological quality bias for all outcomes in most studies ranged from low to moderate levels due to their limited sample size, selection bias, and publication bias (Supplementary Table 1).

### 3.2. Primary and Secondary Outcomes

**Figure 2:**
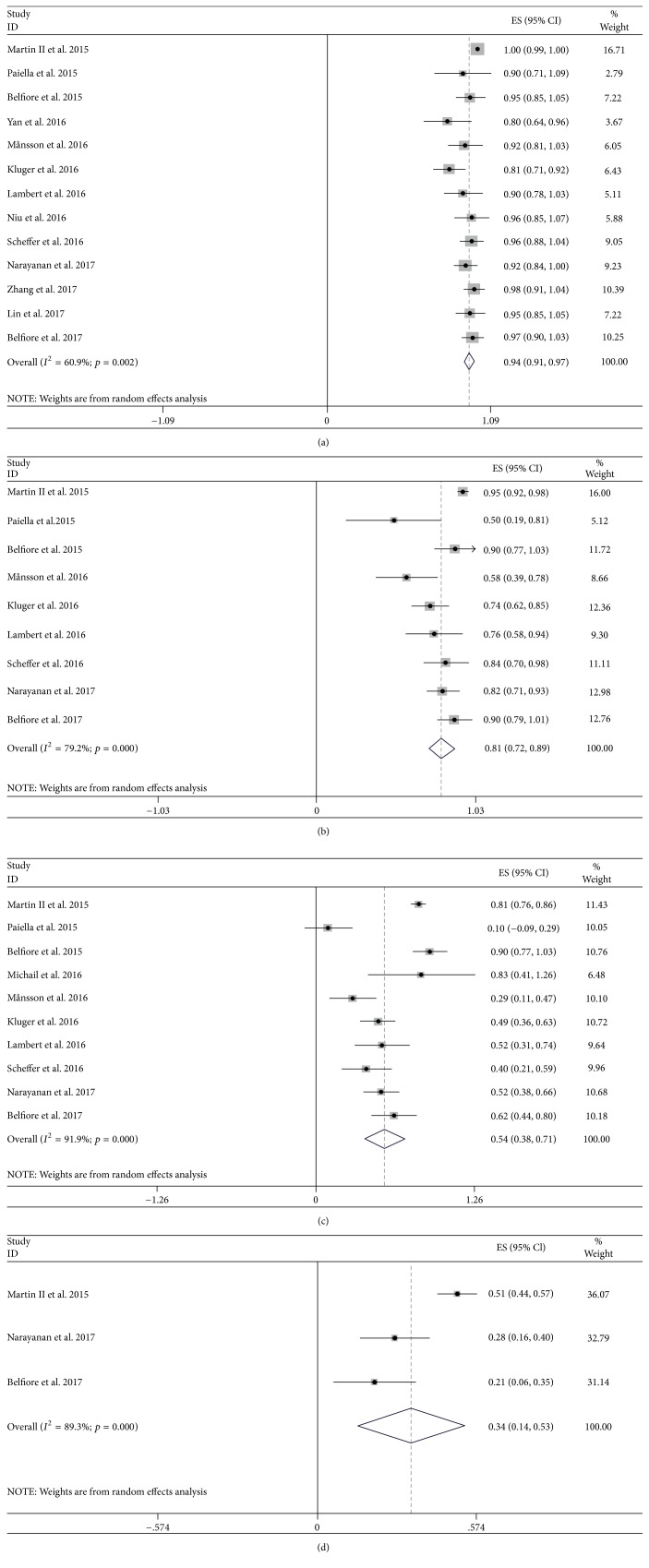
Forest plot of overall survival among studies targeting the effect of IRE therapy in patients with PC at 3 (a), 6 (b), 12 (c), and 24 (d) months.

**Figure 3:**
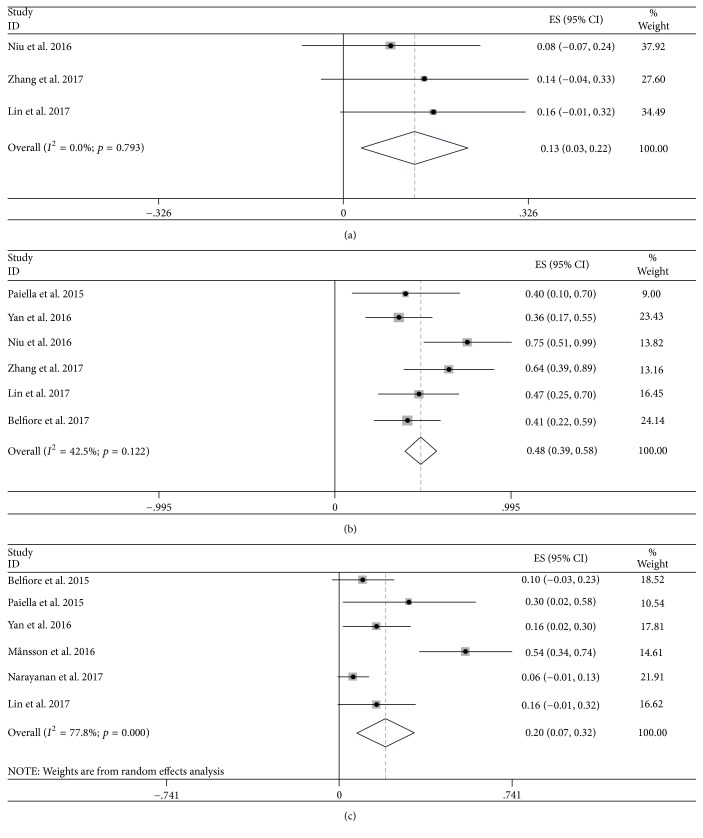
Forest plot of the pooled prevalence data of complete response at 2 months (a), partial response at 3 months (b), and progression at 3 months (c) among studies targeting the effect of IRE therapy in patients with PC.

As shown in Supplementary Table 1 and Figures 2 and 3, the primary outcomes showed that the pooled prevalence estimates of overall survival were 94.1% (95% CI: 90.7–97.5), 80.9% (95% CI: 72.5–89.4), 54.5% (95% CI: 38.3–70.6), and 33.8% (95% CI: 14.2–53.5) at 3, 6, 12, and 24 months, with obvious evidence of between-study heterogeneity (*I*^2^ = 60.9% and *p* = 0.002; *I*^2^ = 79.2% and *p* < 0.001; *I*^2^ = 91.9% and *p* < 0.001; *I*^2^ = 89.3% and *p* < 0.001). And the pooled prevalence data of CR at 2 months, PR at 3 months, progression at 3 months were 12.5% (95% CI: 2.9–22.2), 48.5% (95% CI: 39.4–57.6), and 19.7% (95% CI: 7.3–32.2) (Supplementary Figures 1–12), in which the heterogeneity between studies was acceptable except CR (*I*^2^ = 0 and *p* = 0.793; *I*^2^ = 42.5% and *p* = 0.122; *I*^2^ = 77.8% and *p* < 0.001). Furthermore, among 4 studies, there were detectable publication bias stratified by the overall survival through Egger test (*t* = −5.77 and *p* < 0.001; *t* = −5.28 and *p* < 0.001; *t* = −2.43 and *p* = 0.041; *t* = −24.73 and *p* = 0.026).

The secondary outcomes showed that the pooled incidence values of post-IRE complications were abscess 6.6% (95% CI: 0.2–13), fistula 10.6% (95% CI: 2.5–18.7), pain 33.5% (95% CI: 14.5–52.5), infection 16.1% (95% CI: 3.9–28.4), thrombosis 4.9% (95% CI: 1.2–8.5), pancreatitis 7.2% (95% CI: 3.1–11.2), bleeding 4.2% (95% CI: −0.5–8.9), cholangitis 4.2% (95% CI: −0.5–8.9), nausea 9.6% (95% CI: 4.4–14.8), biliary obstruction 13.8% (95% CI: 4.2–23.3), chest tightness 7.6% (95% CI: 0.5–14.6), and hypoglycemia 5.9% (95% CI: −0.4–12.2), in which between-study heterogeneity was found in pain subgroup (*I*^2^ = 74.8% and *p* = 0.008). During the publication bias assessment in post-IRE complications, it showed that nausea and hypoglycemia were significant (*t* = 7.63 and *p* = 0.005; *t* = 34.22 and *p* = 0.019).

Additionally, sensitivity analysis stratified by primary and secondary outcomes indicated that there was no individual article significantly changing the overall prevalence estimate, by repeating meta-analysis after deleting each study.

## 4. Discussion

This systematic review is the most comprehensive evaluation of clinical benefits in IRE treatment of patients with PC. Regarding the outcome of IRE, we carried out 15 separate articles and found that overall survival values were 94.1%, 80.9%, 54.5%, and 33.8% at 3, 6, 9, 12, and 24 months, and the pooled incidence data of CR at 2 month, PR at 3 month, progression at 3 month were 12.5%, 48.5%, and 19.7%, respectively. A low mortality in IRE could result from main gastrointestinal surgery and other serious diseases in these patients [26]. However, if RFA were applied in PC, the results showed complication rates (15%) and a progression-free survival rate of 22% during the follow-up of 12 months [35]. Previous studies showed that the proportion of 7-month survival in PC patients in resection and no resection group was 53.19% versus 70.40% [36], and the 12-month overall survival in the radiation and nonradiation group was 43% and 29%, respectively [37]. In fact, as an emerging technique, IRE was usually conducted percutaneously or by open surgical or laparoscopic access, which was superior in unresectable lesions. In addition, it was usually used if the lesions were not suitable for other thermal ablations such as MWA, RFA, and cryoablation due to the proximity to sensitive structures such as important blood vessels and gastrointestinal tract. IRE may induce cell apoptosis in the case of supporting the intact cellular structure, and thus PC treatment was enabled, and it was well-known that the pancreas was deep seated in the retroperitoneum. In short, our study showed that IRE had been favorable in prolonging survival, and IRE of PC could be safer than other thermal ablation procedures [38].

Another interesting finding from this study was that post-IRE complications remained a crucial concern during the process of IRE for pancreatic cancer. It was reported that the rate of postpercutaneous IRE complications ranged from 0 to 37% [9, 27, 31], most of which were shown self-limiting and curable with relatively low incidence, such as abscess (7%), nausea (10%), chest tightness (8%), and hypoglycemia (6%). In RFA for PC, postprocedural adverse events occurred in pancreatic fistula (4%–18.8%), gastrointestinal hemorrhage (4%–18.8%), thrombosis (4.7%–15.4%), and acute pancreatitis (2%–11.5%) [35, 38–40]. It was reported that some complications like bowel and biliary perforation in RFA and MWA could be directly induced by thermal injury [41]. Previous study showed that IRE increased heating to cause “white zone” thermal coagulation in case of high energy intensity, which should be not negligible and may be related to thermal damage inducing cell death, confirmed by a thick rim of Hsp70, a marker of thermal damage adjacent to the zone of ablation, similar to thermal ablations [42]. It may be that this thermal damage would be advantageous to facilitate the effect of IRE therapy via enlarging the treatment scope. In addition, it was advised that temperature monitoring could be in consideration during the procedure of IRE ablation around vital structures [43]. This finding would be crucial to further study, based on the low rates of complications reported by IRE.

The present study has several limitations explained as follows. First, relatively small samples could partially account for the main heterogeneity. Particularly in primary outcomes, it may result from the variation of limited participants number in each study. Second, historical treatments and individual differences in studies may have effect on the present heterogeneity. Third, this analysis did not differentiate patients for both early and late stages of pancreatic cancer and their percentage of position distribution, which could be involved in this study as confounding factors, but we did not have enough power to perform subgroup analysis. Fourth, the assessment risk of evidence quality using GRADE criteria was subjective, and tests for publication bias should be seriously interpreted given that the results may be overestimated.

## 5. Conclusions

In summary, though evidence of IRE-related injury in the pancreas was relatively low, the potential for damage needed further consideration. This meta-analysis indicated that IRE may be safe and effective in prolonging survival for patients with pancreatic cancers. More pairwise-comparison studies estimating IRE against traditional surgical resection and interventional therapies will be needed to identify that clinical benefits are available.

## Figures and Tables

**Table 1:** Summary of the studies included in the systematic review.

Author	Study period	Design style	Country	Tumor size	Treatment methods	Patients (number of benign thyroid nodules)	Male/female	Age (years)	Follow-up interval (months)	Complication	Imaging methods	Prognosis
Paiella et al. 2015	2011.6–2011.12	Prospective cohort	Italy	30 mm	US-guided percutaneous IRE	10 (10)	5/5	66	7.6	1, abscesses; 5, abdominal and back pain; 1, portal vein thrombosis; 1, peripheral oedema; 1, infection	CT	3, progression disease; 1, pulmonary metastasis; 2, liver metastasis

Martin II et al. 2015	2010.7–2014.10	Prospective cohort	United States	1–7 cm	CT-guided percutaneous IRE	200	101/99	62 (27–88)	29 months	37% complications	CT	58, recurrence; overall progression-free survival: 12.4 (4.4–38.9); time to distant progression: 16.8 (1.3–55)

Belfiore et al. 2015	2013.4–2014.6	Prospective cohort	Italy	93 (39–170) cm3	CT-guided percutaneous IRE	20 (20)	10/10	69 (55–82)	8.55 (3–14)	1 ascites;	CeCT	18, PR; 2, died

Yan et al. 2016	2015.7–2016.6	Retrospective cohort	China	4.2 (2.8–4.9) cm	Open IRE with/without surgery	25 (25)	19/6	58 (49–80)	3	3, pancreatic fistula; 1, acute pancreatitis; 1, delayed gastric emptying; 1, gastrointestinal hemorrhage	CT/MRI	9, PD; 7, SD

Michail et al. 2016	2014.4–2015.3	Retrospective cohort	United Kingdom	12.3 ± 9.3 mm	CT-guided percutaneous IRE	3 (3)	NA	53 ± 20.8	15 ± 3.6	1 mild pancreatitis	CT	3, not recurrence

Månsson et al. 2016	NA	Retrospective cohort	Sweden	27 ± 15.5 cm3	US-guided percutaneous IRE	24	12/12	63.1 ± 8.6	17.9	5, infection; 2, pancreatitis; 1, thrombosis of the superior mesenteric vein	NA	10, recurrence; 13, metastases; mean OS: 17.9 months

Kluger et al. 2016	2012.10–2015.1	Prospective cohort	United States	3.0 (1.7–5.0) cm	NA	50 (53)	31/22	66.5 (60.2–72.0)	8.69	1, duodenal ulceration/perforation	NA	31, recurrence; mean OS: 7.71–12.03 months

Lambert et al. 2016	2012.6–2014.12	Prospective cohort	Czech Republic	38.2 ± 11.5 mm	NA	21	10/11	68.2 ± 8.4	10.2	1, liver abscesses; 1, biliary peritonitis; 1, cholangitis; 1, pancreatic fistula; 1, bleeding; 1, peripancreatic abscess; 1, fistula and abscess in the abdominal wall	CT	Median survival afer IRE was 10.2 months

Vroomen et al. 2016	2014.1–2015.6	Prospective cohort	Netherlands	19 cm3	CT-guided percutaneous IRE	25 (25)	12/13	61 (41–78)	6 (3–17)	1, pancreatitis; 1, duodenal wall ulcer; 3, biliary obstruction; 1, cholangitis	CeCT/CeMRI	5, local recurrence

Niu et al. 2016	2015.7.2–2015.9.30	Prospective cohort	China	4.3 ± 1.9 (2.4–7.4) cm	7 CT-guided percutaneous IRE; 5 open IRE	12 (12)	9/3	55.5 ± 13.8 (49–75)	1	No major complications	CeCT	1, CR; 9, PR; 2, SD

Scheffer et al. 2016	2014.1–2015.6	Prospective cohort	Netherlands	4.0 (3.3–5.0) cm	CT-guided percutaneous IRE	25 (25)	12/13	61 (41–78)	12 (7–16)	1, pancreatitis; 1, hematemesis; 3, biliary obstruction; 1, cholangitis	CeCT	Median event-free survival from IRE: 8 months; median time to local progression from IRE: 12 months

Narayanan et al. 2017	2010.11.1–2015.8.31	Retrospective cohort	United States	3.2 ± 1.3 cm	CT-guided percutaneous IRE	50 (50)	27/23	62.5 (46–91)	27	19, abdominal pain; 6, pancreatitis; 3, thrombosis; 1, sepsis; 1, gastric leak; 3, nausea; 4, emesis; 3, hematoma; 1, fever; 1, constipation; 1, urinary discomfort; 1, back pain; 1, malaise	CT	3, progression; median OS was 27.0 months

Zhang et al. 2017	2015.7–2017	Prospective cohort	China	3.5 (2.0–6.7) cm	CT/US-guided percutaneous IRE	21 (21)	NA	NA	1	1, hypoglycemia, 1, hypokalemia, 1, chest tightness	CT	2, CR; 9, PR; 3, SD

Lin et al. 2017	2016.3–2016.12	Prospective cohort	China	4.51 ± 1.13 cm	CT/US-guided percutaneous IRE	20 (20)	11/9	53	2	7, fever; 4, fatigue; 3, chest distress; 2, chills; 2, nausea; 1, hypoglycemia	CT/US	3, CR; 9, PR; 4, SD; 3, PD

Belfiore et al. 2017	2013–2016	Retrospective cohort	Italy	94 cm3	CT-guided percutaneous IRE	29 (29)	16/13	68.5	29	No major complications	CeCT	22, died; the median OS: 14 months

NA: not available; CR: complete response; PR: partial response; OS: overall survival; IRE: irreversible electroporation; US: ultrasonography; CT: computed tomography; CeCT: contrast-enhanced computed tomography.

## Abbreviations in Order of Appearance

PC: Pancreatic cancer
IRE: Irreversible electroporation
CR: Complete response
PR: Partial response
PRISMA: Preferred Reporting Items for Systematic Reviews and Meta-Analyses
HIFU: High-intensity focused ultrasound
RFA: Radiofrequency ablation
MWA: Microwave ablation
GRADE: Grading of Recommendations Assessment, Development, and Evaluation.

## Supplementary Materials

Supplementary Materials Supplementary Text 1: search strategy. Supplementary Table 1: subgroup analysis and quality assessment of included studies by primary outcomes and secondary outcomes. Supplementary Figure 1: forest plot of the incidence rate ratios of abscess after IRE therapy for patients with PC. Supplementary Figure 2: forest plot of the incidence rate ratios of fistula after IRE therapy for patients with PC. Supplementary Figure 3: forest plot of the incidence rate ratios of pain after IRE therapy for patients with PC. Supplementary Figure 4: forest plot of the incidence rate ratios of infection after IRE therapy for patients with PC. Supplementary Figure 5: forest plot of the incidence rate ratios of thrombosis after IRE therapy for patients with PC. Supplementary Figure 6: forest plot of the incidence rate ratios of pancreatitis after IRE therapy for patients with PC. Supplementary Figure 7: forest plot of the incidence rate ratios of bleeding after IRE therapy for patients with PC. Supplementary Figure 8: forest plot of the incidence rate ratios of cholangitis after IRE therapy for patients with PC. Supplementary Figure 9: forest plot of the incidence rate ratios of nausea after IRE therapy for patients with PC. Supplementary Figure 10: forest plot of the incidence rate ratios of biliary obstruction after IRE therapy for patients with PC. Supplementary Figure 11: forest plot of the incidence rate ratios of chest tightness after IRE therapy for patients with PC. Supplementary Figure 12: forest plot of the incidence rate ratios of hypoglycemia after IRE therapy for patients with PC.
