# Supplementary material for: Irreversible Electroporation in Patients with Pancreatic Cancer: How Important Is the New Weapon?
Source: Biomed Res Int. 2018 Apr 26;2018:5193067. doi: 10.1155/2018/5193067 (PMC5944201; doi:10.1155/2018/5193067)
Supplement: Supplementary Materials — Supplementary Text 1: search strategy. Supplementary Table 1: subgroup analysis and quality assessment of included studies by primary outcomes and secondary outcomes. Supplementary Figure 1: forest plot of the incidence rate ratios of abscess after IRE therapy for patients with PC. Supplementary Figure 2: forest plot of the incidence rate ratios of fistula after IRE therapy for patients with PC. Supplementary Figure 3: forest plot of the incidence rate ratios of pain after IRE therapy for patients with PC. Supplementary Figure 4: forest plot of the incidence rate ratios of infection after IRE therapy for patients with PC. Supplementary Figure 5: forest plot of the incidence rate ratios of thrombosis after IRE therapy for patients with PC. Supplementary Figure 6: forest plot of the incidence rate ratios of pancreatitis after IRE therapy for patients with PC. Supplementary Figure 7: forest plot of the incidence rate ratios of bleeding after IRE therapy for patients with PC. Supplementary Figure 8: forest plot of the incidence rate ratios of cholangitis after IRE therapy for patients with PC. Supplementary Figure 9: forest plot of the incidence rate ratios of nausea after IRE therapy for patients with PC. Supplementary Figure 10: forest plot of the incidence rate ratios of biliary obstruction after IRE therapy for patients with PC. Supplementary Figure 11: forest plot of the incidence rate ratios of chest tightness after IRE therapy for patients with PC. Supplementary Figure 12: forest plot of the incidence rate ratios of hypoglycemia after IRE therapy for patients with PC. [file 5193067.f1.zip › 5193067.f1.doc]

Supplementary Table 1. Subgroup analysis and quality assessment of included studies by primary outcomes and secondary outcomes

| Subgroup | Number of studies | Quality of the evidence  (GRADE) | ES(95%CI) | Heterogeneity test | |  |  | Publication bias | |
| --- | --- | --- | --- | --- | --- | --- | --- | --- | --- |
|  |  |  |  | *Q* | *τ^2^* | *I^2^*(%) | *p* | *t* | *p* |
| Primary outcomes |  |  |  |  |  |  |  |  |  |
| Overall survival |  |  |  |  |  |  |  |  |  |
| 3 month | 13 | Moderate | 94.1(90.7-97.5) | 30.69 | 0.0018 | 60.9 | 0.002 | -5.77 | <0.001 |
| 6 month | 9 | Moderate | 80.9(72.5-89.4) | 38.54 | 0.0114 | 79.2 | <0.001 | -5.28 | <0.001 |
| 12 month | 10 | Moderate | 54.5(38.3-70.6) | 111.47 | 0.0589 | 91.9 | <0.001 | -2.43 | 0.041 |
| 24 month | 3 | Low | 33.8(14.2-53.5) | 18.65 | 0.0266 | 89.3 | <0.001 | -24.73 | 0.026 |
| CR |  |  |  |  |  |  |  |  |  |
| 2 month | 3 | Low | 12.5(2.9-22.2) | 0.46 | NA | 0 | 0.793 | 0.72 | 0.601 |
| PR |  |  |  |  |  |  |  |  |  |
| 3 month | 6 | Low | 48.5(39.4-57.6) | 8.69 | NA | 42.5 | 0.122 | 1.13 | 0.323 |
| Progression |  |  |  |  |  |  |  |  |  |
| 3 month | 6 | Low | 19.7(7.3-32.2) | 84.77 | 0.0378 | 77.8 | <0.001 | 1.09 | 0.319 |
| Secondary outcomes |  |  |  |  |  |  |  |  |  |
| Abscess | 3 | Low | 6.6(0.2-13) | 1.58 | NA | 0 | 0.453 | 1.74 | 0.333 |
| Fistula | 3 | Low | 10.6(2.5-18.7) | 0.08 | NA | 0 | 0.961 | -0.22 | 0.86 |
| Pain | 4 | Low | 33.5(14.5-52.5) | 11.91 | 0.0261 | 74.8 | 0.008 | 0.98 | 0.431 |
| Infection | 2 | Low | 16.1(3.9-28.4) | 0.74 | NA | 0 | 0.39 | NA | NA |
| Thrombosis | 5 | Low | 4.9(1.2-8.5) | 0.53 | NA | 0 | 0.97 | 1.2 | 0.315 |
| Pancreatitis | 6 | Low | 7.2(3.1-11.2) | 3.93 | NA | 0 | 0.559 | 2.09 | 0.105 |
| Bleeding | 3 | Low | 4.2(-0.5-8.9) | 0.02 | NA | 0 | 0.99 | NA | NA |
| Cholangitis | 3 | Low | 4.2(-0.5-8.9) | 0.02 | NA | 0 | 0.99 | NA | NA |
| Nausea | 5 | Low | 9.6(4.4-14.8) | 4.95 | NA | 19.2 | 0.292 | 7.63 | 0.005 |
| Biliary obstruction | 2 | Low | 13.8(4.2-23.3) | 0.17 | NA | 0 | 0.683 | NA | NA |
| Chest tightness | 3 | Low | 7.6(0.5-14.6) | 1.24 | NA | 0 | 0.538 | 1.6 | 0.355 |
| Hypoglycemia | 3 | Very low | 5.9(-0.4-12.2) | 1.1 | NA | 0 | 0.578 | 34.22 | 0.019 |

GRADE: Grading of Recommendations Assessment, Development and Evaluation;

ES = estimate;

CR=complete response;

PR=partial response;

NA=not available

In this study, heterogeneity was estimated by the index of *Q*, *t^2^*, and *I^2^* statistics. *Q* value represents random error; *t^2^* value represents the variation between studies; *I^2^* value represents individual variation accounting for the total heterogeneity.
