# Supplementary material for: Irreversible Electroporation in Patients with Pancreatic Cancer: How Important Is the New Weapon?
Source: Biomed Res Int. 2018 Apr 26;2018:5193067. doi: 10.1155/2018/5193067 (PMC5944201; doi:10.1155/2018/5193067)
Supplement: Supplementary Materials — Supplementary Text 1: search strategy. Supplementary Table 1: subgroup analysis and quality assessment of included studies by primary outcomes and secondary outcomes. Supplementary Figure 1: forest plot of the incidence rate ratios of abscess after IRE therapy for patients with PC. Supplementary Figure 2: forest plot of the incidence rate ratios of fistula after IRE therapy for patients with PC. Supplementary Figure 3: forest plot of the incidence rate ratios of pain after IRE therapy for patients with PC. Supplementary Figure 4: forest plot of the incidence rate ratios of infection after IRE therapy for patients with PC. Supplementary Figure 5: forest plot of the incidence rate ratios of thrombosis after IRE therapy for patients with PC. Supplementary Figure 6: forest plot of the incidence rate ratios of pancreatitis after IRE therapy for patients with PC. Supplementary Figure 7: forest plot of the incidence rate ratios of bleeding after IRE therapy for patients with PC. Supplementary Figure 8: forest plot of the incidence rate ratios of cholangitis after IRE therapy for patients with PC. Supplementary Figure 9: forest plot of the incidence rate ratios of nausea after IRE therapy for patients with PC. Supplementary Figure 10: forest plot of the incidence rate ratios of biliary obstruction after IRE therapy for patients with PC. Supplementary Figure 11: forest plot of the incidence rate ratios of chest tightness after IRE therapy for patients with PC. Supplementary Figure 12: forest plot of the incidence rate ratios of hypoglycemia after IRE therapy for patients with PC. [file 5193067.f1.zip › 5193067.f2.doc]

**Search strategy:**

Pubmed

1. ‘irreversible electroporation’ OR IRE OR ‘nanoknife’
2. pancrea*
3. #1 AND #2
4. ‘irreversible electroporation’[Mesh]
5. ‘pancreas’ [Mesh]
6. #4 AND #5
7. #3 OR #6

Embase

1. ‘irreversible electroporation’:ab,ti
2. ‘IRE’:ab,ti
3. ‘nanoknife’:ab,ti
4. #1 OR #2 OR #3
5. ‘pancrea*’:ab,ti
6. #4 AND #5

Scoups

1. TITLE-ABS-KEY (‘irreversible electroporation’)
2. TITLE-ABS-KEY ( ‘IRE’ )
3. TITLE-ABS-KEY (‘nanoknife’ )
4. #1 OR #2 OR #3
5. TITLE-ABS-KEY (‘pancrea*’)
6. #4 AND #5

Web of science

1. TS=(irreversible electroporation)
2. TS=( IRE)
3. TS=(nanoknife)
4. #1 OR #2 OR #3
5. TS=(pancreas)
6. #4 AND #5
7. TI=(irreversible electroporation)
8. TI=( IRE)
9. TI=( nanoknife)
10. #7 OR #8 OR #9
11. TI=(pancrea*)
12. #10 AND #11
13. #6 OR #12

Cochrane library

1. ‘irreversible electroporation’ OR IRE OR ‘nanoknife’

2. pancrea*

3. #1 and #2

4. MeSH descriptor: [electroporation] explode all trees

5. MeSH descriptor: [pancreas] explode all trees

6. #4 and #5

7. #3 OR #6
